# Supplementary material for: Microplastic detection and remediation through efficient interfacial solar evaporation for immaculate water production
Source: Nat Commun. 2024 Jul 19;15:6081. doi: 10.1038/s41467-024-50421-x (PMC11271572; doi:10.1038/s41467-024-50421-x)
Supplement: Supplementary file 1 — Supplementary Information [file 41467_2024_50421_MOESM1_ESM.pdf]

Supplementary Information for

Microplastic Detection and Remediation through Efficient Interfacial Solar Evaporation for Immaculate Water Production

Zhen Yu,[1,2] Yang Li,[2] Yaoxin Zhang,[3] Ping Xu,[4] Chade Lv,[4] Wulong Li,[5] Bushra Maryam,[2] Xianhua Liu,[2,*] Swee Ching Tan[1,*]

[1] Department of Materials Science and Engineering, National University of Singapore, Singapore 117574, P. R. Singapore

[2] School of Environmental Science and Engineering, Tianjin University, Tianjin 300072, P. R. China

[3] China-UK Low Carbon College, Shanghai Jiao Tong University, Shanghai 201306, P. R. China

[4] MIIT Key Laboratory of Critical Materials Technology for New Energy Conversion and Storage, School of Chemistry and Chemical Engineering, Harbin Institute of Technology, Harbin 150001, P. R. China

[5] School of Electrical and Electronic Engineering, Nanyang Technological University, Singapore 639798, P. R. Singapore

Correspondence: lxh@tju.edu.cn; msetansc@nus.edu.sg

1 Supplementary Methods

1.1 Materials and reagents

Commercial carbon felt (CF), polystyrene (PS, 0.5~80 μm) polymethyl methacrylate (PMMA, 100~600 nm), polyvinyl chloride (PVC, 1~6.5 μm), and polyhydroxybutyrate (PHB, 2~74 μm) were bought from the Alibaba Platform (China). Fluorescence-labeled PS dispersion (500 nm, 1 μm, and 2 μm, spherical shape) was purchased from Aladdin Reagents Co., Ltd. (China). Polyethylenimine (PEI, Mw ~800) was purchased from Sigma Aldrich. All other chemicals were analytical grade and purchased from Sinopharm Chemical Reagent. All materials and reagents were directly used without further purification.

1.2 Characterization

The morphology was observed by the scanning electron microscope (SEM, ZEISS Gemini SEM 300, Germany). The Fourier-transform infrared (FT-IR, Frontier Optica PerkinElmer, America) spectra were obtained at room temperature from 4000 to 500 cm^{-1}. The contact angle was recorded by the dynamic contact angle tester (OCA20, Germany). The light absorption was measured by a UV-Vis-NIR spectrophotometer (UV-3101, Japan) equipped with an integrating sphere. The element distribution was recorded by the X-ray photoelectron spectroscopy (XPS, PHI Quantera SXM spectrometer, Germany), using a monochromatic Al Kα X-ray source. The ion concentrations in water samples (condensed water and original seawater) were measured by an inductively coupled plasma spectrometer (ICP-AES, ICP-6000, UK). The number of MPs in the water samples was counted by a flow cytometer (Exflow-104, DAKEWE, China). The identification of MPs in seawater was conducted by a Raman confocal microscopy (Alpha 300R system, WITec Company) with laser wavelengths of 532 nm and 785 nm).

1.3 Extraction and detection of MPs

MPs in water samples were extracted through a simple digestion-filtration method, which could be divided into the following steps[1, 2]: (1) Water samples were collected by the stainless-steel containers and filtered through stainless steel mesh screens of 5

mm, 1 mm, 330 μm, 250 μm, and 125 μm in sequence. (2) After filtration, the as-obtained solution was treated by vacuum filtration (the pore size of the filter membrane is 80 μm), and the filtrate was treated by vacuum filtration (the pore size of the filter membrane is 2 μm) again. (3) Then the filter membrane was transferred to a 500 mL glass beaker and digested by 180 mL KOH (10%, m/v) and 20 mL H_2O_2 (30%) mixed solution under 60 °C for 24 h. After removing the filter membrane from the digestion solution, the digestion solution was treated by vacuum filtration (the pore size of the filter membrane is 2 μm). (4) The as-obtained filter membrane was transferred into a glass dish and dried naturally. The processed MPs samples could be observed in the above filter membrane by using an optical microscope (Leica M165FC).

The extraction process of MPs in the soil was similar to that in the water[3, 4]. The sampled soil was washed with saturated NaCl solution, and then the resulting supernatant is stored. The MPs sample in the soil could be obtained by repeating the above (1)~(4) operations on the supernatant.

MPs in the air were sampled using air samplers (JH-6120, Qingdao Jingcheng Co., Ltd., China)[5]. The as-obtained filter membrane was washed with deionized water. The MPs sample in the air could be obtained by repeating the above (1)~(4) operations on the washing liquid.

1.4 Outdoor experiments

Outdoor experiments were conducted in a homemade device in Tianjin (China). The experiment was conducted in seawater from Bohai Sea. The condensed water and the original seawater were measured by 3D-EEM fluorescence spectra (JASCO FP-6500, Japan). MPs in water samples before and after ISEP treatment were extracted through a simple digestion filtration method[1, 2]. The actual removal performance of ISEP on MPs was studied by using optical counting methods.

1.5 DFT calculation

Density functional theory (DFT) calculations were carried out by using the Gaussian 09 software package1. Gaussian 09 software package1 Geometry optimization was carried out at the B3LYP method with a 6-31+G(d,p) basis set. The description of van der Waals interactions was improved using Grimme's empirical

dispersion (GD3) correction. Single-point energy calculations were performed at the M06-2x/Def2-TZVP level.

1.6 The estimation for the regeneration time of ISEP in an outdoor scene

Before estimation, two issues need to be further clarified:

(1) ISEP has the same adsorption capacity to MPs outdoors and indoors

(2) This estimation process ignored the effect of environmental factors such as temperature and humidity.

The regeneration time of ISEP in an outdoor scene was calculated by **Equation (S1)**:

$$T_o = \frac{C_i}{C_o} T_i \tag{S1}$$

Where T_o was the regeneration time of ISEP in an outdoor scene (h); T_i was the regeneration time of ISEP in an indoor scene (60 h); C_i was the concentration of MPs in the indoor experimentes (69687 item m^{-3}); C_o was the concentration of MPs in the real seawater (0.2 ~ 351 item m^{-3}).

1.7 Rapid joule heat treatment on adsorbed PS

The upcycling of PS in CF-PEI using the fast joule heating technique is conducted on the classic sealed tube reactor in an inert atmosphere. After adsorbed saturation to PS, CF-PEI is dried in an oven and then connected to the electrical power source in the quartz tube reactor. Before the reaction, adequate N_2 is pumped through the reactor during the whole reaction. After pumping N_2 for at least 10 min, a current pulse of 60 A is applied to the CF-PEI to decompose the adsorbed PS in 4 s. The released gas was captured in a 1 L gas bag and analyzed by gas chromatography (GC, FULI INSTRRUMENTS GC9790 Plus, China).

2 Supplementary Note: COMSOL Simulations

A transient model was employed to study the adsorption process of the CF-PEI under solar irradiation and dark conditions, respectively[6].

Before simulations, we made the following assumptions:

(1) The classical heat and mass transfer schemes such as the continuity equation were valid under this scale;

(2) Neglecting the effect of temperature on adsorption kinetics.

A 2D geometric model was established as follows.

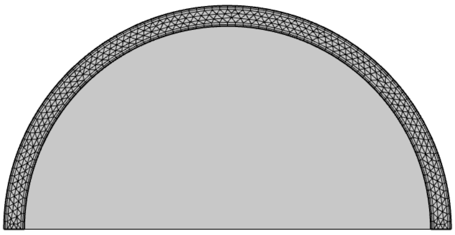

Conservation of momentum in porous materials was described with Brinkman equation:

$$\frac{\mu}{\theta_P}\mathbf{u}=-\nabla P+\nabla\frac{1}{Por}\left[\mu(\nabla\mathbf{u}+(\nabla\mathbf{u})^T)-\frac{2}{3}\mu(\nabla\mathbf{u})I\right] \quad \text{(S2)}$$

Where μ was the dynamic viscosity of the solution (mPa s); $\mathbf{u}$ was the inlet water velocity (m s^{-1}), which was calculated based on the measured evaporation rate under 1 sun or in dark conditions; *Por* was the effective porosity of CF-PEI (%); I is the unit matrix.

The mass transfer of water and MPs here involved three parts: advection, diffusion, and adsorption. The advection and diffusion process were described as follows:

$$\mathbf{S}=\frac{\partial c}{\partial t}+\nabla\cdot(\mathbf{J}+\mathbf{u}c) \quad \text{(S3)}$$

$$\mathbf{J}=-D\nabla c \quad \text{(S4)}$$

Where $\mathbf{S}$ represented the mass source due to water evaporation (m s^{-1}); c was the concentration (wt%, converted from mol L^{-3}); D was the diffusion coefficient (m^2 s^{-1}).

The adsorption process was described as follows:

$$e_a\frac{\partial^2\mathbf{u}}{\partial t^2}+d_a\frac{\partial\mathbf{u}}{\partial t}+\nabla_T\cdot\Gamma=R \quad \text{(S5)}$$

$$\nabla_T=(\mathbf{I}-\mathbf{nn}^T)[\frac{\partial}{\partial x},\frac{\partial}{\partial y}] \quad \text{(S6)}$$

$$R=k_1\cdot c\cdot(Gs-\mathbf{u})-k_2\cdot\mathbf{u} \quad \text{(S7)}$$

Where e_a was the quality coefficient (s); d_a was the damping factor; k_1 and k_2 were the positive reaction rate constant (m^3 (mol s)$^{-1}$) and reverse reaction rate constant (s^{-1}), respectively; Gs was the active site concentration (mol m^{-2}).

4 Supplementary Figures and Tables

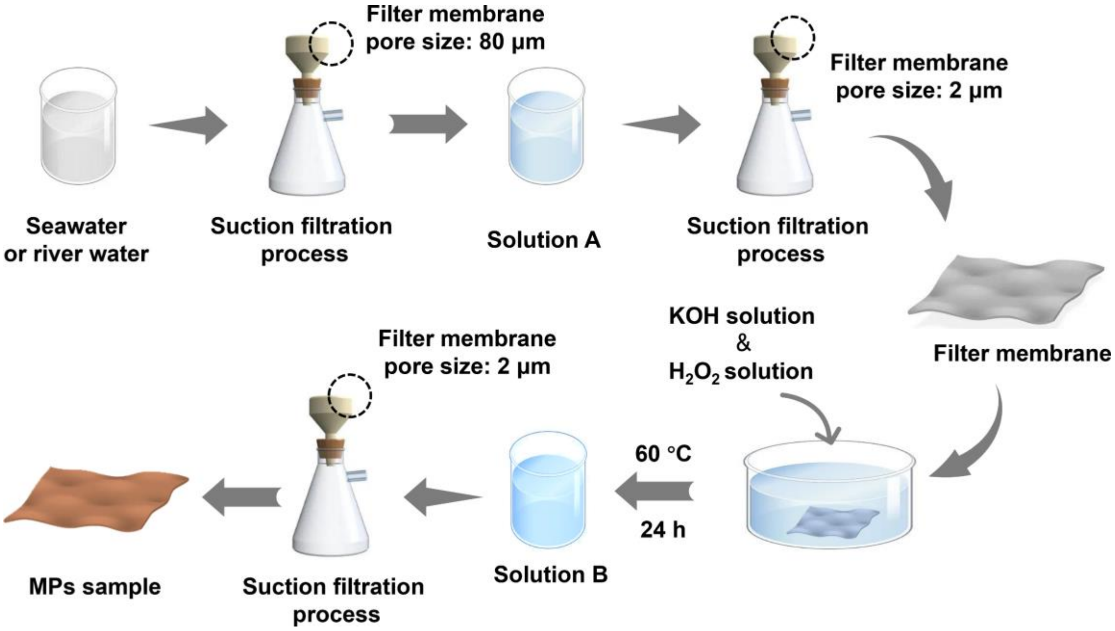

Supplementary Fig. 1 | Schematic diagram of the digestion-filtration method.

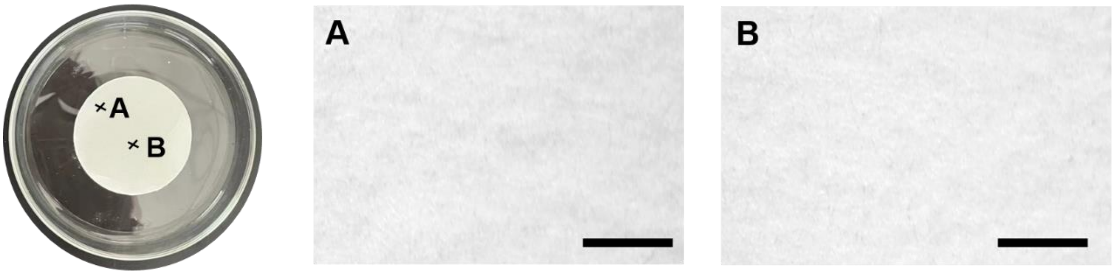

Supplementary Fig. 2 | Microscopic images of the pristine filter membrane. Scale bar: 100 μm.

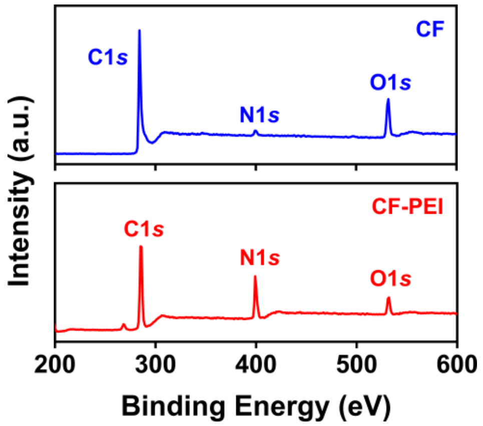

Supplementary Fig. 3 | XPS spectra of CF and CF-PEI.

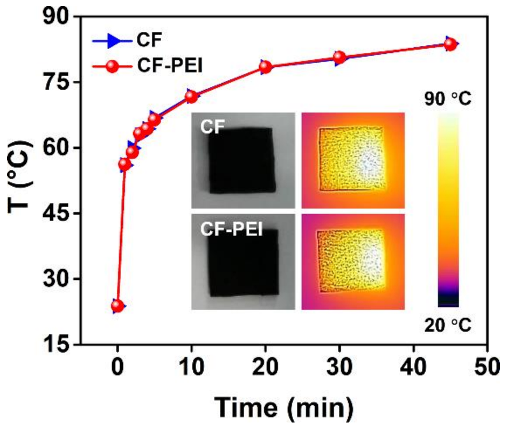

Supplementary Fig. 4 | The temperature rising curves of CF-PEI and CF in a dry state under 1 sun. Inset: The digital photos and IR photos of CF and CF-PEI after irradiation for 45 min.

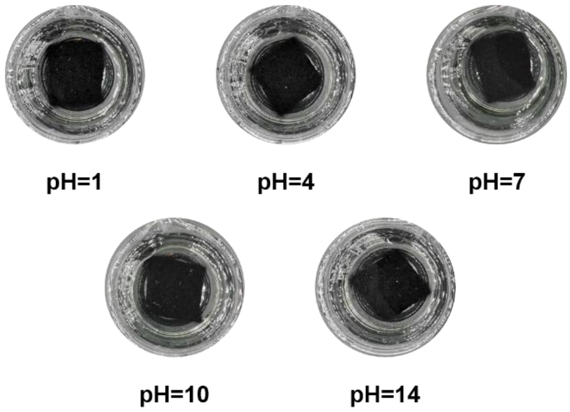

Supplementary Fig. 5 | The digital photos of CF-PEI in the solution with varying pH.

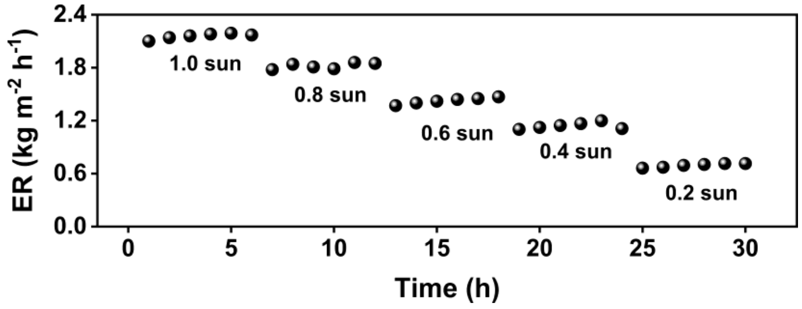

Supplementary Fig. 6 | The evaporation rate of 3D-ISEP in pure water under low solar fluxes.

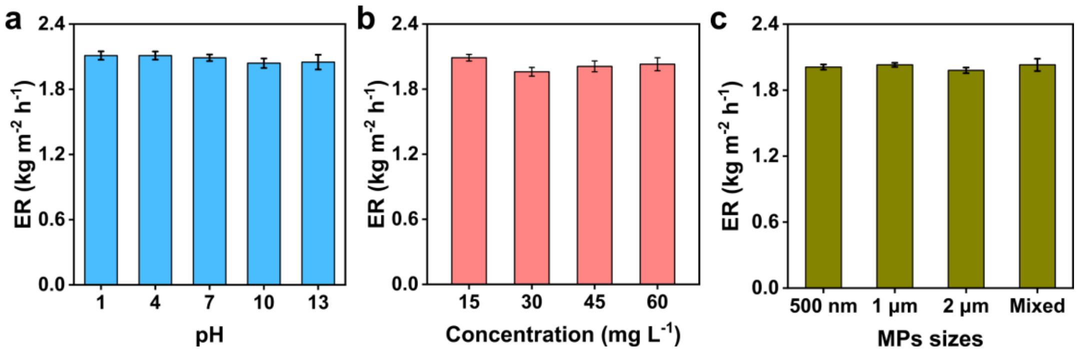

Supplementary Fig. 7 | The evaporation rate of 3D-ISEP under 1 sun in various conditions: (**a**) Different pH of MPs solution. Initial conditions: MPs concentration is 15 mg L^{-1} and the particle size of MPs is 500 nm; (**b**) Different MPs concentrations. Initial conditions: the pH of MPs solution is 7, and the particle size of MPs is 500 nm; (**c**) Different MPs sizes. Initial conditions: MPs concentration is 15 mg L^{-1} and the pH of MPs solution is 7. Error bars represent the standard deviations from three-time measurements.

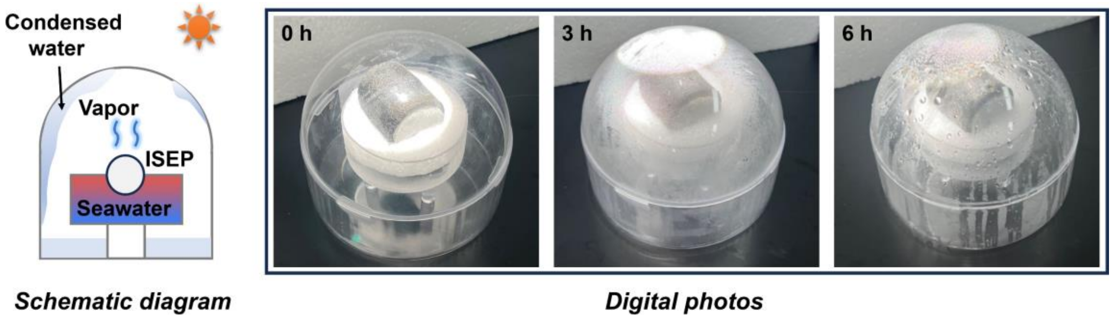

Supplementary Fig. 8 | The schematic and digital photos of the homemade device for collecting the condensed water produced by ISEP. During the initial 6 hours, a significant amount of water mist formed on the top of the device and gradually accumulated at the bottom due to gravity. Condensed water could then be collected from the bottom of this device for further analysis.

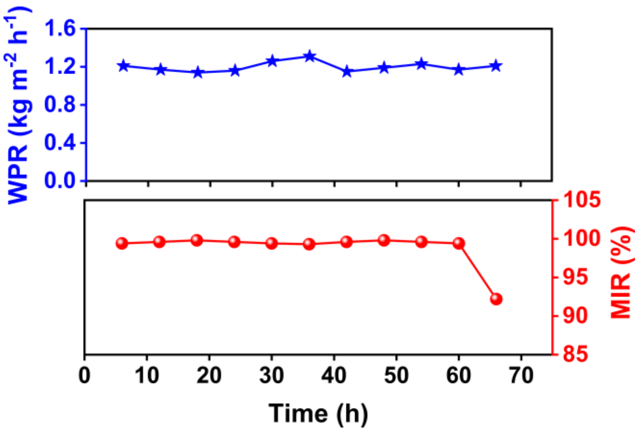

Supplementary Fig. 9 | The water production rate (WPR) and MPs interception ratio (MIR) in the continuous 66 h under 1 sun.

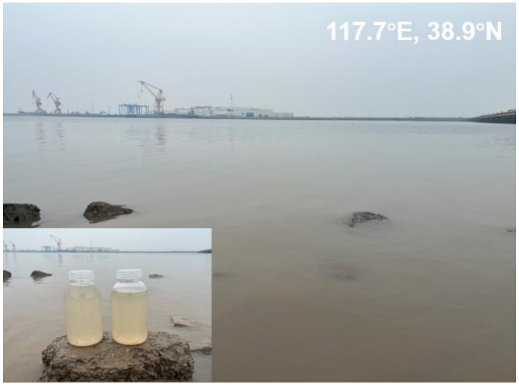

Supplementary Fig. 10 | Sampling points of seawater used in the outdoor experiments.

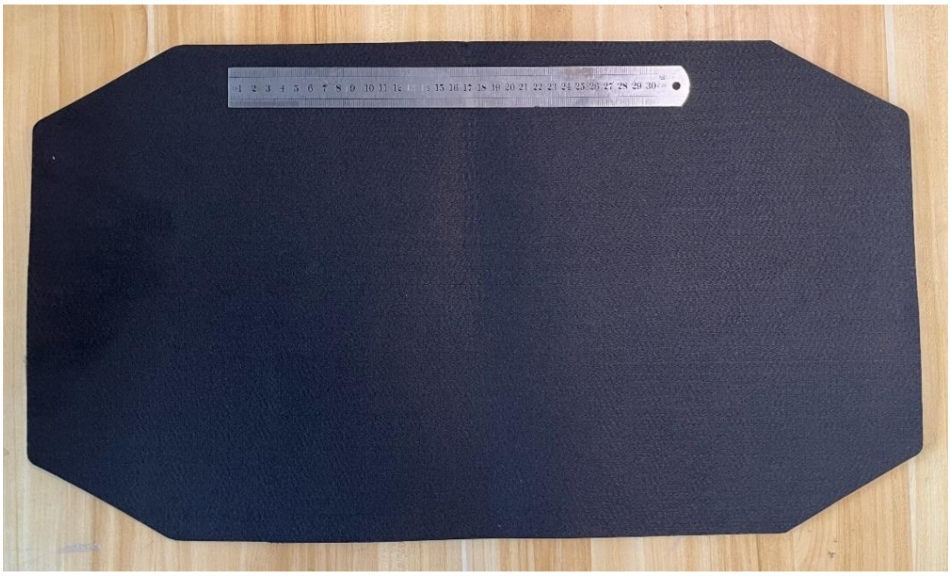

Supplementary Fig. 11 | The digital photo of large-size CF-PEI.

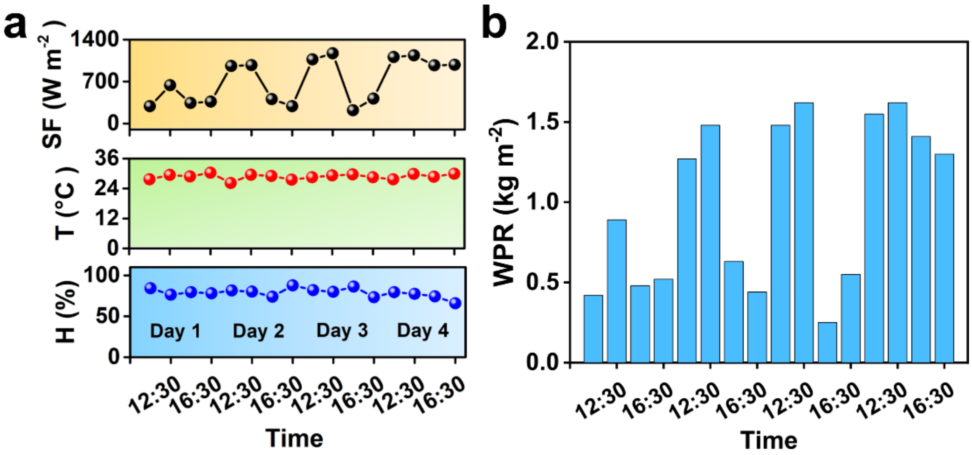

Supplementary Fig. 12 | Outdoor performance of 3D-ISEP. (**a**) Weather conditions in the outdoor experiments; (**b**) The water production rate (WPR) in the outdoor experiments.

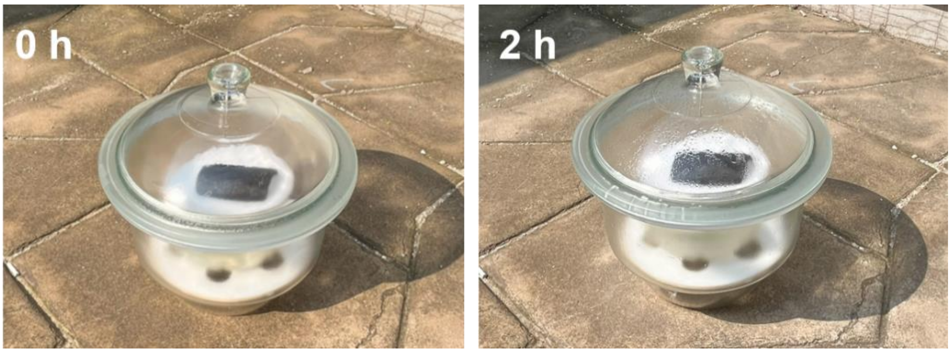

Supplementary Fig. 13 | The digital photos of the outdoor device.

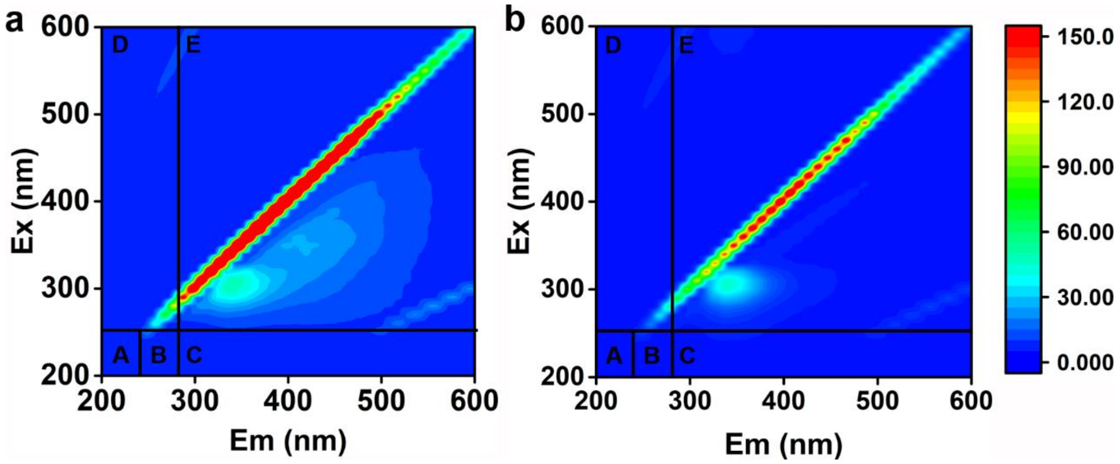

Supplementary Fig. 14 | 3D-EEM images. (**a**) The original seawater; (**b**) The condensed water.

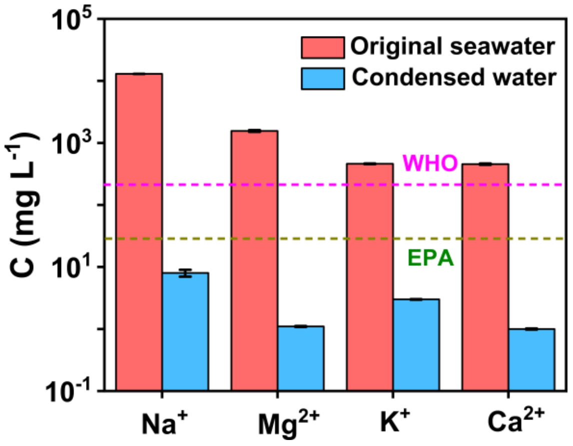

Supplementary Fig. 15 | The ion concentrations of the original seawater and condensed water produced by ISEP. Error bars represent the standard deviations from three-time measurements.

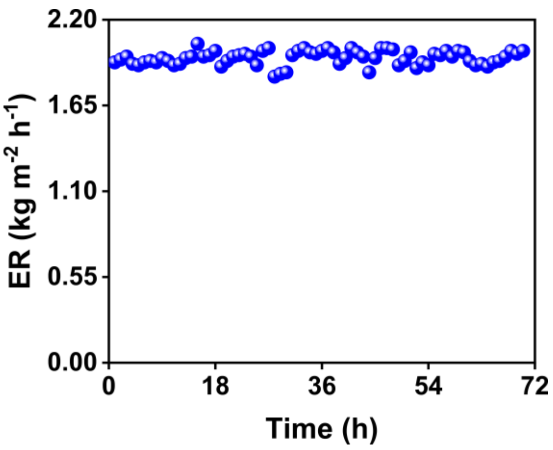

Supplementary Fig. 16 | The evaporation rate of ISEP based on CF-PEI in the long-term desalination experiments under 1 sun.

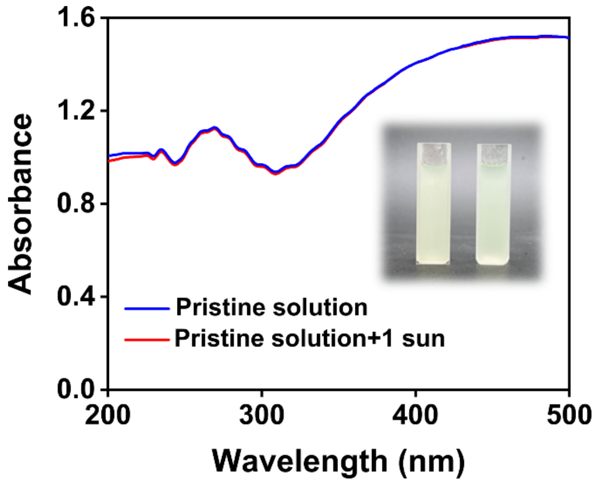

Supplementary Fig. 17 | UV/Vis spectra of the PS solution before and after irradiation for 48 h under 1 sun. Inset: The digital photos of PS solution before and after irradiation for 48 h under 1 sun.

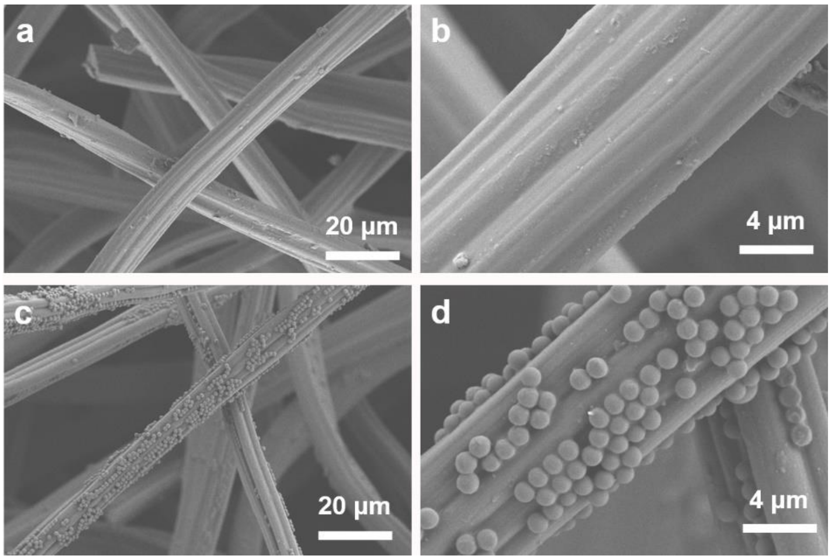

Supplementary Fig. 18 | SEM images. (**a**) CF-PEI of ISEP before absorbing MPs; (**b**) Fiber in CF-PEI of ISEP before absorbing MPs; (**c**) CF-PEI of ISEP after absorbing MPs; (**d**) Fiber in CF-PEI of ISEP after absorbing MPs.

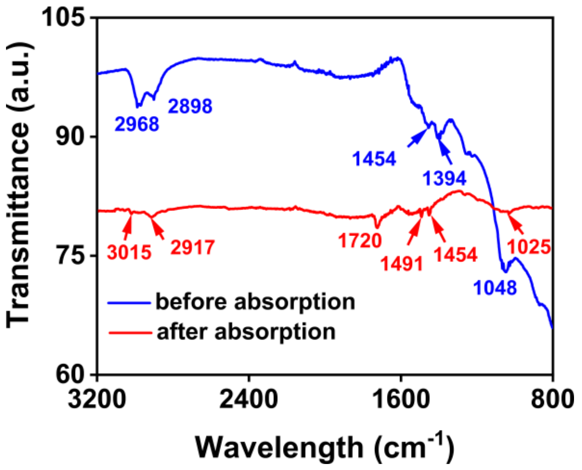

Supplementary Fig. 19 | FT-IR spectra of CF-PEI before and after absorbing PS. Some novel peaks appeared. Specifically, the new peaks at 3015, 1720, and 1025 cm^{-1} are related to C=O, $-CH_2$, and C-O, respectively[7, 8]. The peak appearing at 2917 cm^{-1} corresponds to the stretching vibration of C-H, and the peaks appearing at 1491 and 1454 cm^{-1} are related to the vibration of the benzene ring[9, 10, 11].

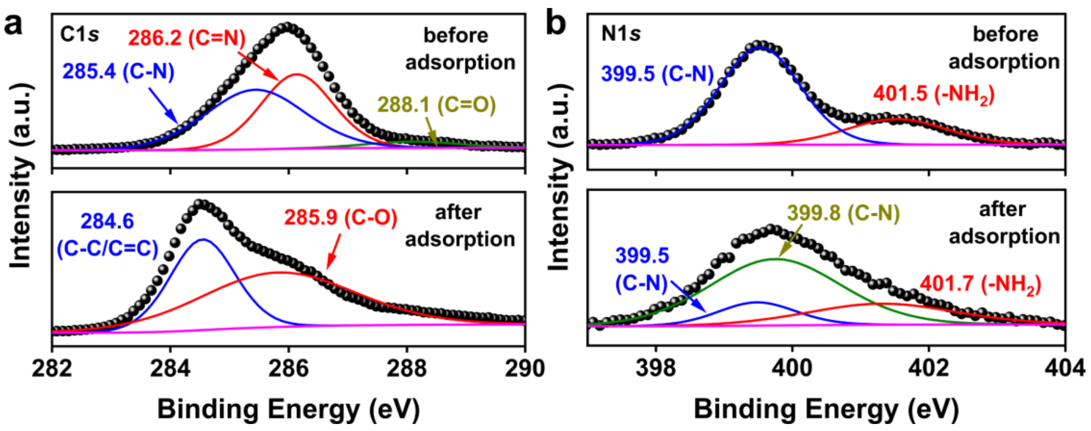

Supplementary Fig. 20 | XPS spectra of CF-PEI before and after absorbing PS. (**a**) C1*s*; (**b**) N1*s*.

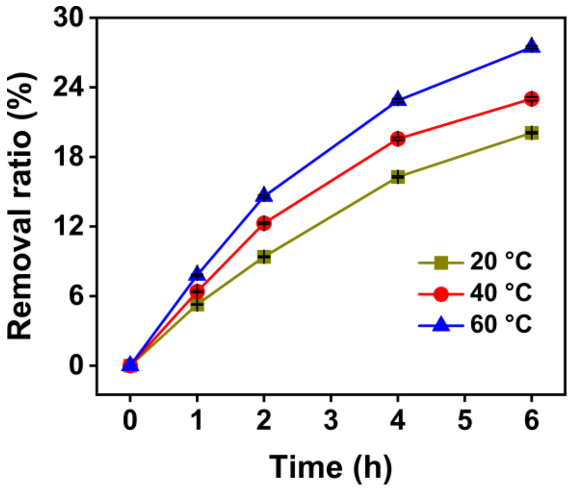

Supplementary Fig. 21 | The MPs removal ratio by ISEP based on CF-PEI under different temperatures in dark. Error bars represent the standard deviations from three-time measurements.

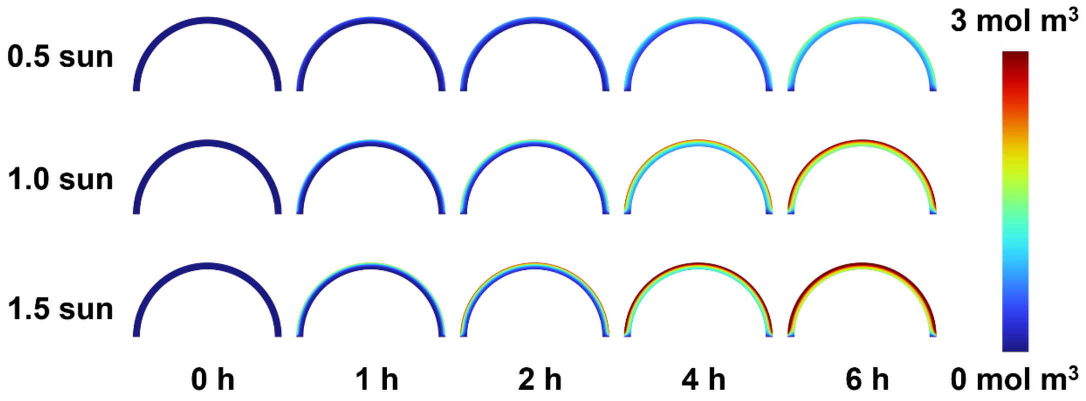

Supplementary Fig. 22 | The MPs enrichment performance by ISEP based on CF-PEI under different solar flux simulated by COMSOL.

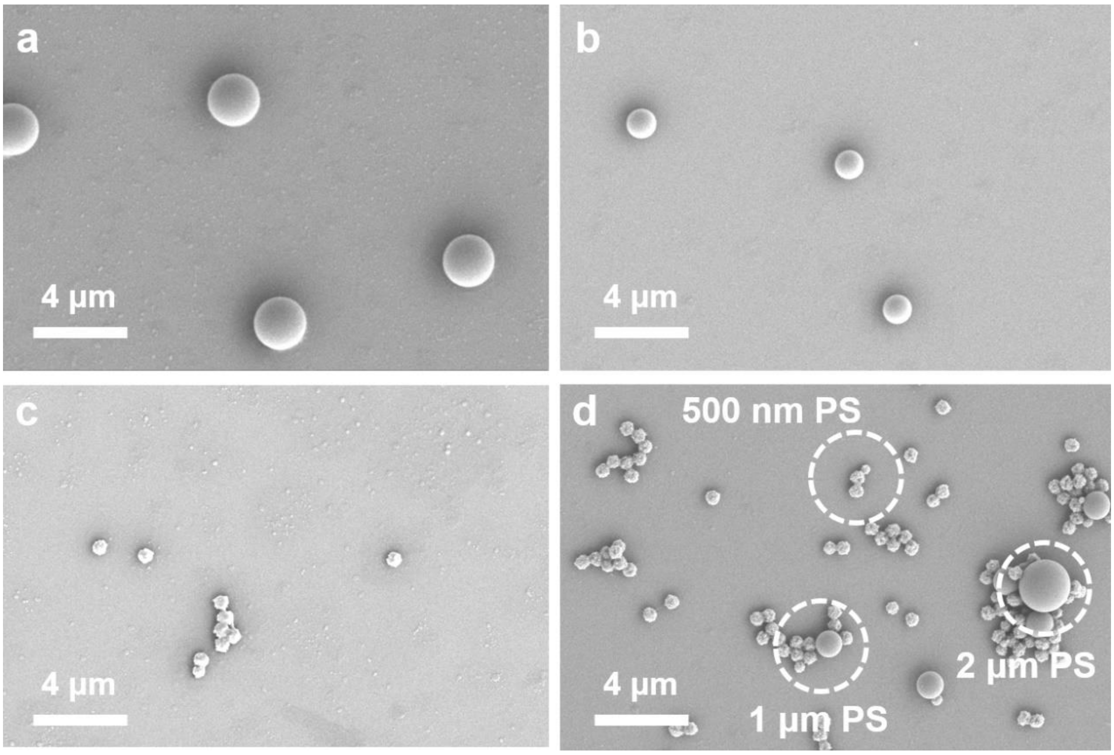

Supplementary Fig. 23 | SEM images of PS with different particle sizes. (**a**) 2 μm; (**b**) 1 μm; (**c**) 500 nm; and (**d**) Mixed size.

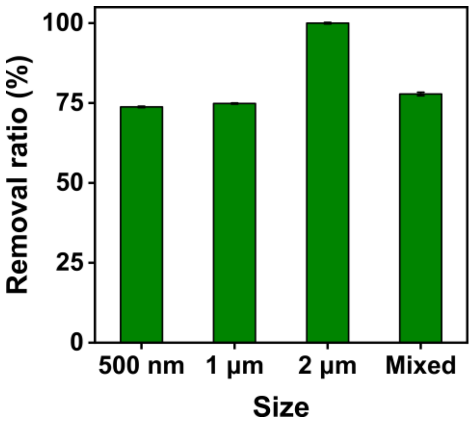

Supplementary Fig. 24 | The removal ratio by ISEP on PS with different particle sizes. Initial conditions: the pH of MPs solution is 7, and MPs concentration is 15 mg L^{-1}. Error bars represent the standard deviations from three-time measurements.

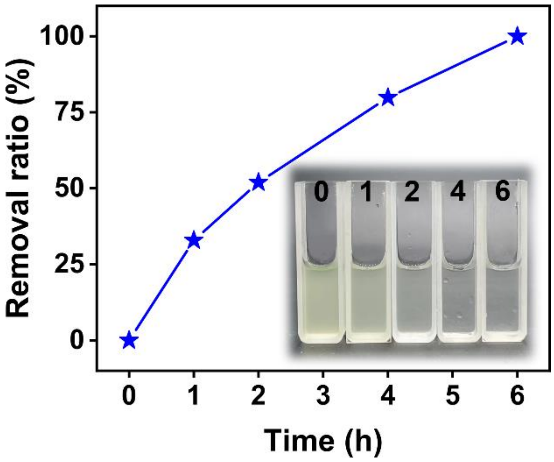

Supplementary Fig. 25 | The removal ratio of PS by ISEP. Initial conditions: the particle size of MPs is 500 nm; the pH of MPs solution is 7, and MPs concentration is 15 mg L^{-1}. Inset: The digital photos of MPs solution on different removal times.

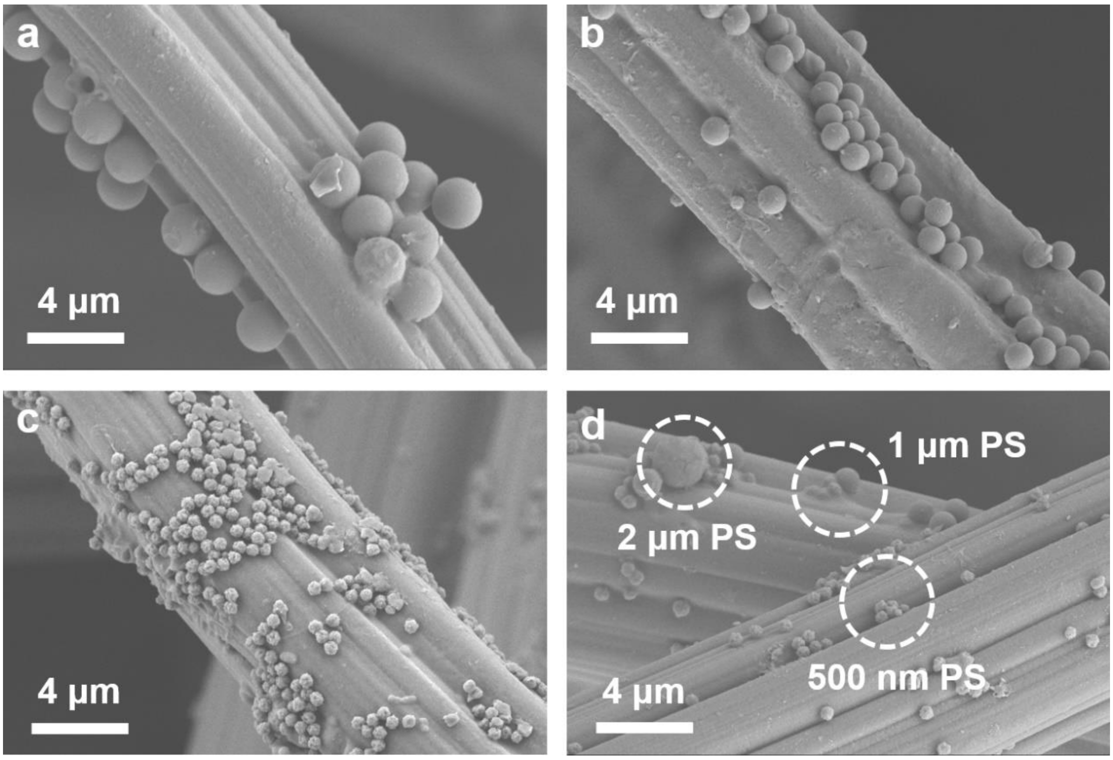

Supplementary Fig. 26 | SEM images of fiber in CF-PEI of ISEP after absorbing MPs with different particle sizes. (**a**) 2 μm; (**b**) 1 μm; (**c**) 500 nm; and (**d**) Mixed size.

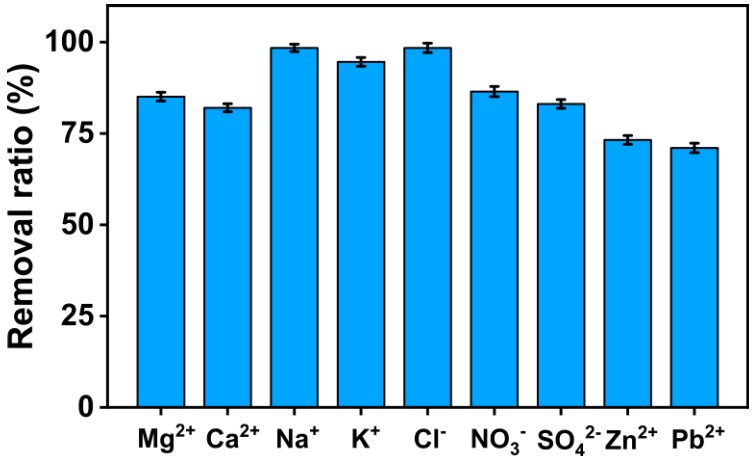

Supplementary Fig. 27 | The removal ratio of PS by ISEP in the presence of various metal ions. Initial conditions: the pH of MPs solution is 7, MPs concentration is 15 mg L^{-1}, and the particle size of MPs is 500 nm. Error bars represent the standard deviations from three-time measurements.

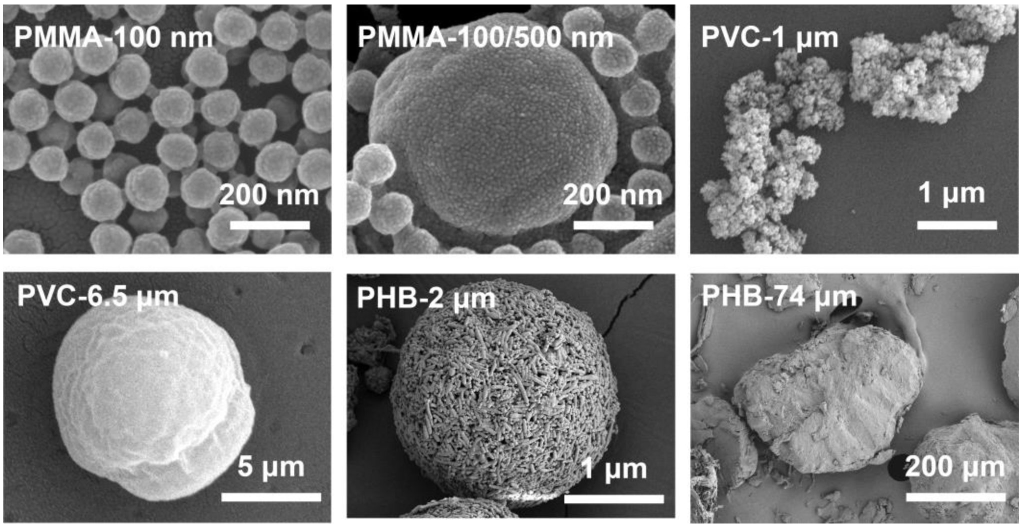

Supplementary Fig. 28 | SEM images of MPs with different types, shapes, and sizes.

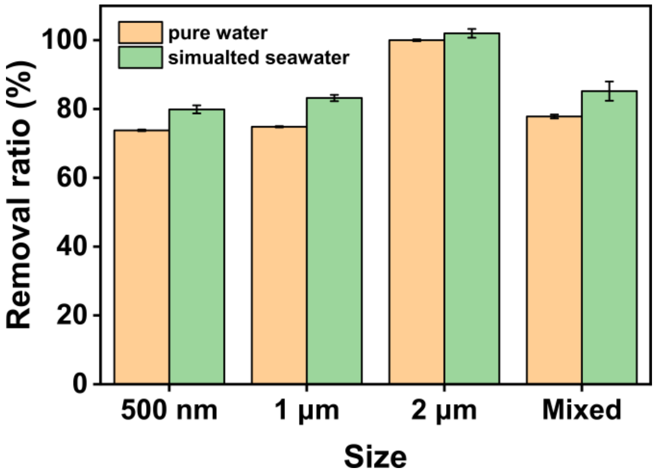

Supplementary Fig. 29 | The removal ratio of PS by ISEP in the PS solution prepared by pure water and simulated seawater. Initial conditions: the pH of MPs solution is 7, MPs concentration is 15 mg L^{-1}. The simulated seawater is prepared by a typical procedure[12]. Error bars represent the standard deviations from three-time measurements.

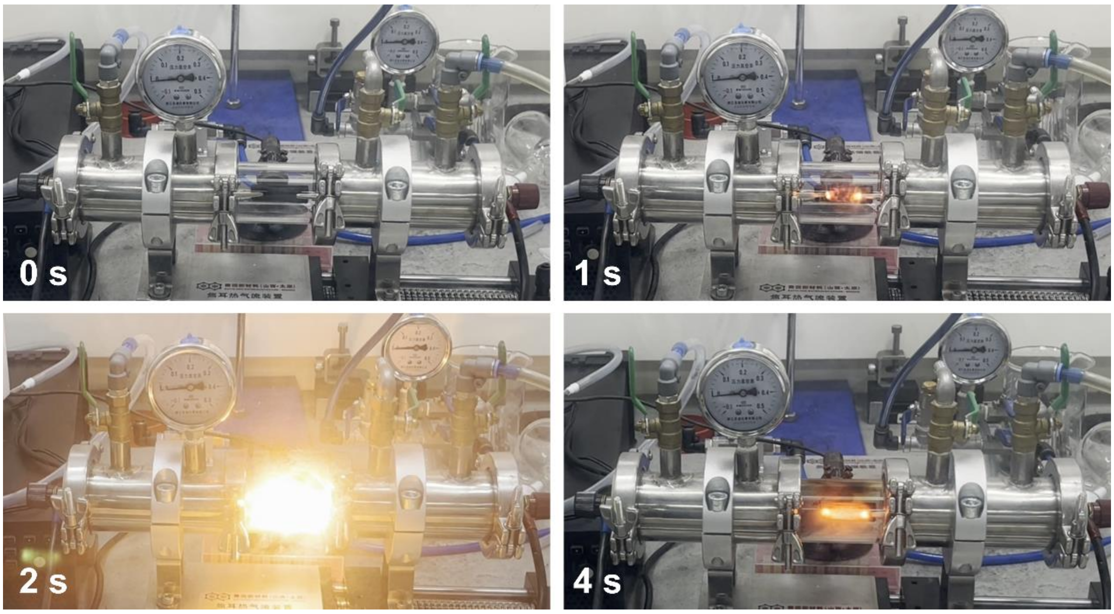

Supplementary Fig. 30 | The digital photos of the upcycle and conversion of PS absorbed by CF-PEI by fast flash joule heating technology.

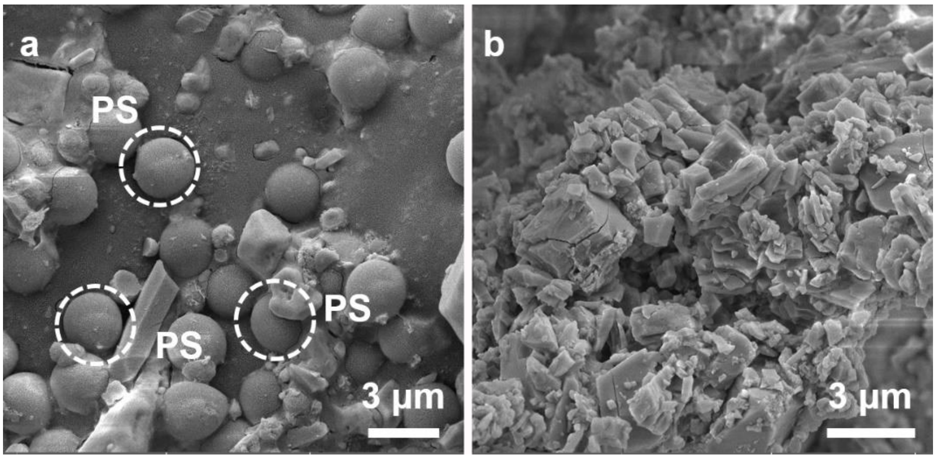

Supplementary Fig. 31 | SEM images of the sea salts produced from the simulated seawater containing PS (**a**) before and (**b**) after treatment by ISEP.

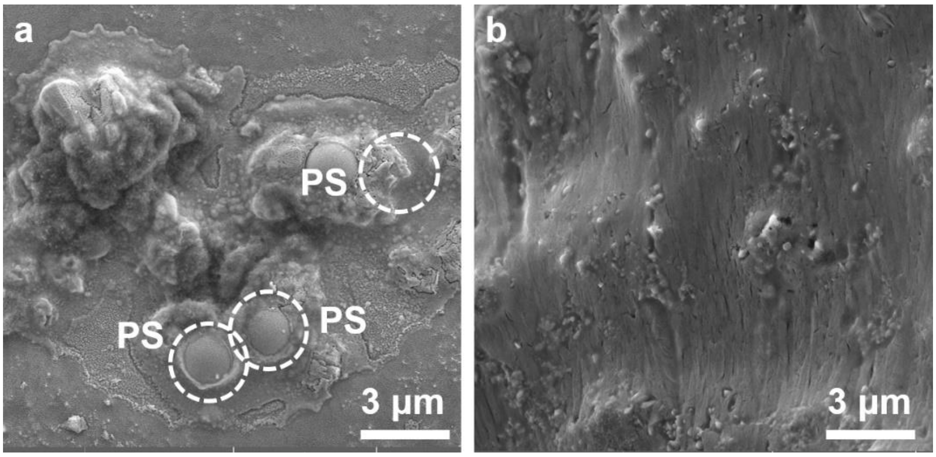

Supplementary Fig. 32 | SEM images of the NaOH produced from the simulated seawater containing PS (**a**) before and (**b**) after treatment by ISEP.

Supplementary Table 1 | Zeta potentials (pH ~7.0) of the different MPs.

MPs	Zeta potential (mV)
PMMA	-26.1
PS	-27.2
PVC	-29.5
PHB	+19.5

Supplementary Table 2 | Zeta potentials (pH ~7.0) of CF and CF-PEI before and after adsorbing different MPs.

Adsorbents	Zeta potential (mV)
CF	-32.2
CF-PEI	+31.2
CF-PEI after adsorption of PMMA	-4.1
CF-PEI after adsorption of PS	-4.8
CF-PEI after adsorption of PVC	-6.3
CF-PEI after adsorption of PHB	+37.6

Supplementary Table 3 | The MPs removal ratio of previously reported adsorbents, ISEP fabricated by these adsorbents in dark and under 1 sun. Error bars represent the standard deviations from three-time measurements.

Adsorbents	Reported MPs removal ratio	MPs removal ratio by ISEP in dark	MPs removal ratio by ISEP under 1 sun
Magnetic nano-Fe_3O_4 (Metal-based)[13]	32 ± 1.2% (30 min)	27 ± 2.3% (30 min)	88 ± 2.1% (30 min)
Magnetic carbon nanotubes (Carbon-based)[14]	14 ± 2.8% (60 min)	11 ± 1.2% (60 min)	72 ± 1.5% (60 min)
Cu-Ni carbon material (Hybrid-based)[15]	40 ± 3.6% (120 min)	34 ± 2.8% (120 min)	90 ± 2.1% (120 min)

Supplementary Table 4 | The gas product obtained by upgrading PS (absorbed by CF-PEI) through fast joule heating technology.

**Gas composition**	**Conversion ratio (%)**	**Gas composition**	**Conversion ratio (%)**
**H_2**	**93.2**	CH_4	2.0
C_2H_4	4.7	C_2H_6	0.03
C_3H_6	0.06	C_3H_8	0.03

Supplementary references

1. Prata JC, da Costa JP, Duarte AC, Rocha-Santos T. Methods for sampling and detection of microplastics in water and sediment: a critical review. *TrAC, Trends Anal Chem* **110**, 150-159 (2019).
2. Lusher A, Welden N, Sobral P, Cole M. Sampling, isolating and identifying microplastics ingested by fish and invertebrates. In: *Analysis of nanoplastics and microplastics in food*). CRC Press (2020).
3. Zhang S, Yang X, Gertsen H, Peters P, Salánki T, Geissen V. A simple method for the extraction and identification of light density microplastics from soil. *Science of The Total Environment* **616-617**, 1056-1065 (2018).
4. He D, Luo Y, Lu S, Liu M, Song Y, Lei L. Microplastics in soils: Analytical methods, pollution characteristics and ecological risks. *TrAC Trends in Analytical Chemistry* **109**, 163-172 (2018).
5. Habibi N, Uddin S, Fowler SW, Behbehani M. Microplastics in the atmosphere: A review. *J Environ Expo Assess* **1**, 10.20517 (2022).
6. Xu J*, et al.* Solar-driven interfacial desalination for simultaneous freshwater and salt generation. *Desalination* **484**, 114423 (2020).
7. Wu J, Chen T, Luo X, Han D, Wang Z, Wu J. TG/FTIR analysis on co-pyrolysis behavior of PE, PVC and PS. *Waste Management* **34**, 676-682 (2014).
8. Gan B, Bilek M, Kondyurin A, Mizuno K, McKenzie D. Etching and structural changes in nitrogen plasma immersion ion implanted polystyrene films. *Nuclear Instruments and Methods in Physics Research Section B: Beam Interactions with Materials and Atoms* **247**, 254-260 (2006).
9. Subramani M, Sepperumal U. FTIR analysis of bacterial mediated chemical changes in polystyrene foam. *Ann Biol Res* **7**, 55-61 (2016).
10. Battulga B, Kawahigashi M, Oyuntsetseg B. Characterization of biofilms formed on polystyrene microplastics (PS-MPs) on the shore of the Tuul River, Mongolia. *Environ Res* **212**, 113329 (2022).
11. Liu Y, He Z, Uchimiya M. Comparison of biochar formation from various

agricultural by-products using FTIR spectroscopy. *Modern Applied Science* **9**, 246 (2015).

12. Yu Z, Gu R, Tian Y, Xie P, Jin B, Cheng S. Enhanced Interfacial Solar Evaporation through Formation of Micro-Meniscuses and Microdroplets to Reduce Evaporation Enthalpy. *Advanced Functional Materials* **32**, 2108586 (2022).
13. Shi X, Zhang X, Gao W, Zhang Y, He D. Removal of microplastics from water by magnetic nano-Fe_3O_4. *Science of The Total Environment* **802**, 149838 (2022).
14. Tang Y, Zhang S, Su Y, Wu D, Zhao Y, Xie B. Removal of microplastics from aqueous solutions by magnetic carbon nanotubes. *Chemical Engineering Journal* **406**, 126804 (2021).
15. Zhou G*, et al.* Removal of polystyrene nanoplastics from water by CuNi carbon material: The role of adsorption. *Science of The Total Environment* **820**, 153190 (2022).
