# Supplementary material for: Microplastic detection and remediation through efficient interfacial solar evaporation for immaculate water production
Source: Nat Commun. 2024 Jul 19;15:6081. doi: 10.1038/s41467-024-50421-x (PMC11271572; doi:10.1038/s41467-024-50421-x)
Supplement: Supplementary file 3 — Reporting Summary [file 41467_2024_50421_MOESM3_ESM.pdf]

nature portfolio

Corresponding author(s): Swee Ching TAN

Last updated by author(s): Jun 24, 2024

Reporting Summary

Nature Portfolio wishes to improve the reproducibility of the work that we publish. This form provides structure for consistency and transparency in reporting. For further information on Nature Portfolio policies, see our Editorial Policies and the Editorial Policy Checklist.

Statistics

For all statistical analyses, confirm that the following items are present in the figure legend, table legend, main text, or Methods section.

n/a	Confirmed	
x	☐	The exact sample size (*n*) for each experimental group/condition, given as a discrete number and unit of measurement
x	☐	A statement on whether measurements were taken from distinct samples or whether the same sample was measured repeatedly
x	☐	The statistical test(s) used AND whether they are one- or two-sided<br>*Only common tests should be described solely by name; describe more complex techniques in the Methods section.*
x	☐	A description of all covariates tested
x	☐	A description of any assumptions or corrections, such as tests of normality and adjustment for multiple comparisons
x	☐	A full description of the statistical parameters including central tendency (e.g. means) or other basic estimates (e.g. regression coefficient) AND variation (e.g. standard deviation) or associated estimates of uncertainty (e.g. confidence intervals)
x	☐	For null hypothesis testing, the test statistic (e.g. *F*, *t*, *r*) with confidence intervals, effect sizes, degrees of freedom and *P* value noted<br>*Give P values as exact values whenever suitable.*
x	☐	For Bayesian analysis, information on the choice of priors and Markov chain Monte Carlo settings
x	☐	For hierarchical and complex designs, identification of the appropriate level for tests and full reporting of outcomes
x	☐	Estimates of effect sizes (e.g. Cohen's *d*, Pearson's *r*), indicating how they were calculated

Our web collection on statistics for biologists contains articles on many of the points above.

Software and code

Policy information about availability of computer code

Data collection	No software was used to collect the data in this study.
Data analysis	We mainly used 'OriginPro Learning Edition' and 'office 2019' to analyse the data. No novel codes were used in the study.

For manuscripts utilizing custom algorithms or software that are central to the research but not yet described in published literature, software must be made available to editors and reviewers. We strongly encourage code deposition in a community repository (e.g. GitHub). See the Nature Portfolio guidelines for submitting code & software for further information.

Data

Policy information about availability of data

All manuscripts must include a data availability statement. This statement should provide the following information, where applicable:

- Accession codes, unique identifiers, or web links for publicly available datasets
- A description of any restrictions on data availability
- For clinical datasets or third party data, please ensure that the statement adheres to our policy

The data that supports the findings of the study are included in the main text and supplementary information files. Raw data can be obtained from the corresponding author upon request.

Research involving human participants, their data, or biological material

Policy information about studies with human participants or human data. See also policy information about sex, gender (identity/presentation), and sexual orientation and race, ethnicity and racism.

Reporting on sex and gender	This issue is not applicable to our study.
Reporting on race, ethnicity, or other socially relevant groupings	This issue is not applicable to our study.
Population characteristics	This issue is not applicableto our study.
Recruitment	This issue is not applicableto our study.
Ethics oversight	This issue is not applicableto our study.

Note that full information on the approval of the study protocol must also be provided in the manuscript.

Field-specific reporting

Please select the one below that is the best fit for your research. If you are not sure, read the appropriate sections before making your selection.

☐ Life sciences ☐ Behavioural & social sciences ☒ Ecological, evolutionary & environmental sciences

For a reference copy of the document with all sections, see nature.com/documents/nr-reporting-summary-flat.pdf

Ecological, evolutionary & environmental sciences study design

All studies must disclose on these points even when the disclosure is negative.

Study description	Microplastics (MPs) pollution and freshwater scarcity have become the most concerning global challenges (Nature 619, 317–322 (2023), Nature 622, 499–506 (2023)). To address these issues, we propose an interfacial solar evaporation platform (ISEP) fabricated by MPs adsorbent, which can boost the MPs removal and simultaneously produce clean water free of MPs.
Research sample	Not applicable. No sampling experiment involved.
Sampling strategy	Not applicable. No sampling experiment involved.
Data collection	All the evaporation data was recorded by Data Logger, and all the MPs removal data was collected manually. Zhen Yu and Yang Li has conduct the above process.
Timing and spatial scale	The data was recodred from 2023.02 to 2023.11.
Data exclusions	For all performance data indicators (such as removal ratios and evaporation rates), we usually conduct more than five experiments. After removing the maximum and minimum values, we average the remaining data. For all spectral data (such as Raman, etc.), we repeat the test for three times and select the appropriate data.
Reproducibility	We repeat all the indoor experiments for over three times, and every experiment was successful.
Randomization	Not applicable. No sampling experiment involved.
Blinding	Not applicable. No sampling experiment involved.

Did the study involve field work? ☐ Yes ☒ No

Reporting for specific materials, systems and methods

We require information from authors about some types of materials, experimental systems and methods used in many studies. Here, indicate whether each material, system or method listed is relevant to your study. If you are not sure if a list item applies to your research, read the appropriate section before selecting a response.

Materials & experimental systems

n/a	Involved in the study	
☒	☐	Antibodies
☒	☐	Eukaryotic cell lines
☒	☐	Palaeontology and archaeology
☒	☐	Animals and other organisms
☒	☐	Clinical data
☒	☐	Dual use research of concern
☐	☒	Plants

Methods

n/a	Involved in the study	
☒	☐	ChIP-seq
☒	☐	Flow cytometry
☒	☐	MRI-based neuroimaging

Dual use research of concern

Policy information about dual use research of concern

Hazards

Could the accidental, deliberate or reckless misuse of agents or technologies generated in the work, or the application of information presented in the manuscript, pose a threat to:

No	Yes	
☒	☐	Public health
☒	☐	National security
☒	☐	Crops and/or livestock
☒	☐	Ecosystems
☒	☐	Any other significant area

Experiments of concern

Does the work involve any of these experiments of concern:

No	Yes	
☒	☐	Demonstrate how to render a vaccine ineffective
☒	☐	Confer resistance to therapeutically useful antibiotics or antiviral agents
☒	☐	Enhance the virulence of a pathogen or render a nonpathogen virulent
☒	☐	Increase transmissibility of a pathogen
☒	☐	Alter the host range of a pathogen
☒	☐	Enable evasion of diagnostic/detection modalities
☒	☐	Enable the weaponization of a biological agent or toxin
☒	☐	Any other potentially harmful combination of experiments and agents

Plants

Seed stocks	We used Orient Caixin Hong Kong (Benih Sawi Bunga 205) to study the accumulation process of microplastics in plants. The seeds of Orient Caixin Hong Kong were bought from Alibaba Co., Ltd. of China
Novel plant genotypes	We did not change the genotypes of the plants.
Authentication	We did not change the genotypes of the plants.
